# Tunable Electrokinetic Motion of Charged Nanoparticles in an Aqueous Solution Using Interdigitated Microelectrodes

**DOI:** 10.3390/nano15201568

**Published:** 2025-10-15

**Authors:** Farshad Rezakhanloo, Yera Ussembayev, Mohammadreza Bahrami, Filip Beunis, Kevin Braeckmans, Ilia Goemaere, Deep Punj, Amin Ahmad, Louis Van der Meeren, Kristiaan Neyts

**Affiliations:** 1 LCP Research Group, Ghent University, Technologiepark 126, 9052 Gent, Belgium; farshad.rezakhanloo@ugent.be (F.R.); yerzhan.ussembayev@ugent.be (Y.U.); mohammadreza.bahrami@ugent.be (M.B.); filip.beunis@ugent.be (F.B.); 2 Center for Nano and Biophotonics, Ghent University, Technologiepark 126, 9052 Gent, Belgium; kevin.braeckmans@ugent.be; 3 Faculty of Pharmaceutical Sciences, Ghent University, Ottergemsesteenweg 460, 9000 Gent, Belgium; iliagoemaere2@hotmail.com (I.G.); deep.punj@ugent.be (D.P.); amin.ahmad@ugent.be (A.A.); 4 Trince Bio, Ottergemsesteenweg Zuid 731, 9000 Gent, Belgium; louis.vandermeeren@trincebio.com; 5 Department of Electronic and Computer Engineering, Hong Kong University of Science and Technology, Clear Water Bay, Kowloon, Hong Kong

**Keywords:** charged particles, particle sorting, micro- and nanoparticles, electrophoresis, dielectrophoresis, electroosmosis

## Abstract

Electrokinetic phenomena offer promising tools for the manipulation of micro- and nanoparticles in liquid media. However, most existing techniques rely on complex configurations and are often limited to particle separation based on large size differences or distinct material properties. Here, we present a simple and tunable method for spatial control and separation of nanoparticles using interdigitated electrodes under AC electric fields. Our approach exploits subtle differences in the electroosmotic and dielectrophoretic responses of particles with small size variations but identical material compositions. By adjusting the frequency and amplitude of the applied voltage, particles can be selectively directed and accumulated at designated regions of the device, enabling precise control over their positioning and segregation. We demonstrate the effectiveness of our method using micro- and nanoparticles composed of the same material, achieving accurate spatial separation based solely on their electrokinetic behavior. This technique offers a low-cost, easily integrable platform for diverse applications, including cell manipulation, water purification, and targeted drug delivery.

## 1. Introduction

Precise manipulation and spatial control of particles in liquids play a vital role in diverse fields such as targeted drug delivery [1,2], environmental remediation [3,4], and colloidal chemistry [5,6]. Traditionally, particle positioning and separation techniques have relied on significant differences in size, composition, or optical properties, often requiring fluorescent labeling or complex microfluidic setups [7,8]. While effective in certain contexts, these approaches are often limited in tunability, biocompatibility, and applicability to particles with subtle physical differences.

Electrokinetic phenomena, such as electrophoresis, dielectrophoresis, and electroosmosis, have emerged as versatile tools for the manipulation of micro- and nanoscale particles suspended in fluidic environments. In particular, alternating current (AC) electric fields applied across microelectrode structures can generate a range of phenomena including dielectrophoresis [9], electrothermal flows [10], and AC electro-osmosis [11,12,13]. These effects have been exploited for many tasks, including particle trapping [14,15,16], transport [17], and assembly [18]. However, despite their promise, most current electrokinetic platforms rely on fixed or complex electrode configurations and static field geometries [19], which inherently limit their operational flexibility and adaptability. Designing such microelectrode patterns is often a challenging and time-consuming process, requiring precise lithographic fabrication to achieve specific field distributions. Consequently, dynamic control over particle positioning or selective manipulation of similar particles remains a significant challenge. Furthermore, effective separation is typically restricted to particles with large disparities in dielectric or geometric properties [20,21], limiting the applicability of these techniques.

In this work, we introduce a simple yet tunable method for controlling the position and separation of charged micro- and nanoparticles using interdigitated microelectrodes under AC electric fields. Our approach enables label-free spatial sorting of particles with similar material composition and minimal size difference based solely on their differential electrokinetic response. By varying the frequency and amplitude of the applied voltage, particles can selectively accumulate in desired regions with high accuracy. The system requires no surface modifications, chemical labels, or complex instrumentation, offering a robust platform for real-time, label-free particle positioning. We further demonstrate the effectiveness of this method using micro- and nanoparticles of identical composition (polystyrene) and evaluate the influence of parameters such as particle size and ion concentration on spatial distribution.

## 2. Materials and Methods

### 2.1. Microchip Fabrication and Sample Preparation

**Figure 1:**
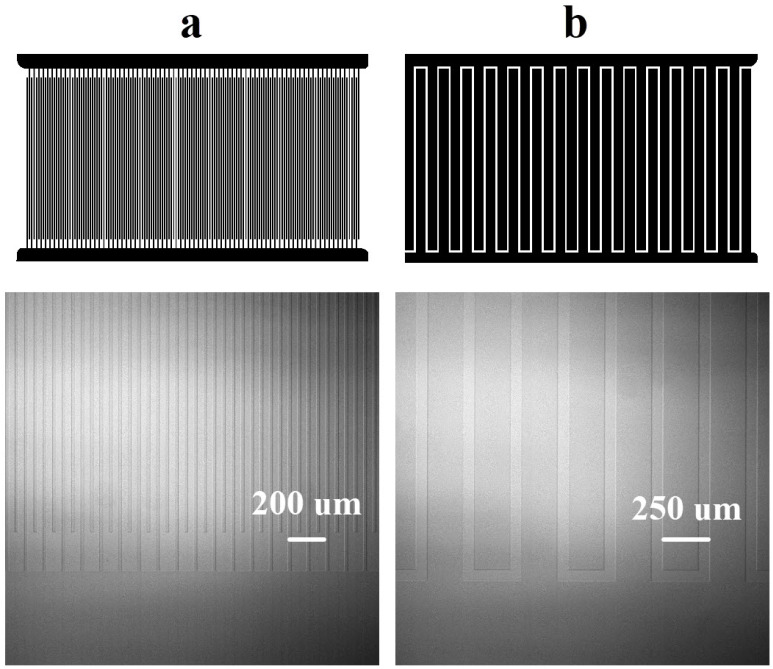
Photolithography masks (**top**) and optical microscopy images of the fabricated interdigitated electrodes (IDE) (**bottom**), (**a**) E25-S25 chip, 74 electrodes with width and separation both equal to 25 mm and (**b**) E200-S50 chip, 15 electrodes with width 200 mm and separation 50 mm. The length of the electrodes is 4000 mm.

The electrodes were designed to generate strong electric fields under low voltage conditions, while avoiding irregularities of the electrode edges and electrodes with an open circuit. Moreover, a non-uniform electric field distribution is targeted, as it enhances the effectiveness of electrokinetic forces such as dielectrophoresis and electroosmosis. In this work, the microchips were fabricated following our previously published protocol [16,22], consisting of a single 1 mm thick glass substrate coated with a conductive, transparent layer of indium tin oxide (ITO) with a few changes in the protocol. Low-resistance ITO-coated glass substrates (5–10 Ω/sq, from Delta Technologies, Loveland, CO, USA) were spin-coated with AZ ECI 3027 photoresist from MicroChemicals (Ulm, Germany) to achieve a 2.5 µm-thick layer. The UV exposure time, using a parallel beam was 26 s. Etching was carried out in a heated mixture of 9 M HCl and 3 wt% FeCl_3_ for 5 min, followed by development in undiluted AZ^®^ 726 MIF, a TMAH-based developer from MicroChemicals. The rest of the protocol remains unchanged. The use of interdigitated electrodes (IDE) is intended to eliminate asymmetries in the system by ensuring a periodic field distribution. To investigate the effects of the geometry of the electrodes on the electrokinetic forces, two different electrode widths *w* and spacing between them *s* were designed and fabricated, referred to as chip E25-S25 and chip E200-S50, respectively. The length *l* of the electrodes is 4 mm. The interdigitated ITO patterns were defined by photolithography using the mask design shown in Figure 1 (top) and wet etching [22], both performed in a cleanroom environment. The optical microscopy images of the resulting microstructures for the two chips are presented in Figure 1 (bottom). During the wet etching process, there is an unwanted “under-etch” phenomenon in which the etchant attacks the substrate laterally, below the photoresist. As a result, the electrodes are about 2–5 μm narrower than the design width.

**Figure 2:**
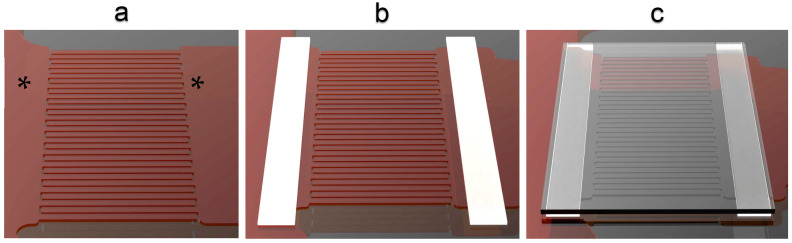
The assembly of microfluidic channels based on the fabricated microchip; (**a**) patterned ITO electrodes on the glass substrate; (**b**) addition of two thin adhesive layers (thickness ~50 mm) as spacers; (**c**) addition of a glass coverslip on top (**c**). Places marked with * are the interdigitated electrodes connections.

Following the electrode fabrication, a microfluidic channel (Figure 2) was assembled using two narrow double-sided adhesive layers (thickness ~50 μm) and a glass coverslip [16,22]. For all measurements, the chip was oriented upside-down, with the electrodes at the upper side, to avoid confounding effects by the gravitational sedimentation of heavier particles to the electrodes. The electrical connection between the voltage source and the chip was established by soldering two metal wires to the ITO areas that connect the interdigitated electrodes. As a model system, we used negatively charged polystyrene particles (“Microbead NIST Traceable Particle”, “Polysciences Inc.”, Warrington, PA, USA) with nominal diameters of 500 nm, 1 μm, and 2 μm. These particles, which are supplied in suspension form (%2.5 *w*/*v*), were suspended in deionized water (resistivity > 1 MOhm.cm) to achieve a concentration of (%2.5 × 10^−3^ *w*/*v*), as specified in the manufacturer’s technical datasheet.

### 2.2. Electric Circuit Model

**Figure 3:**
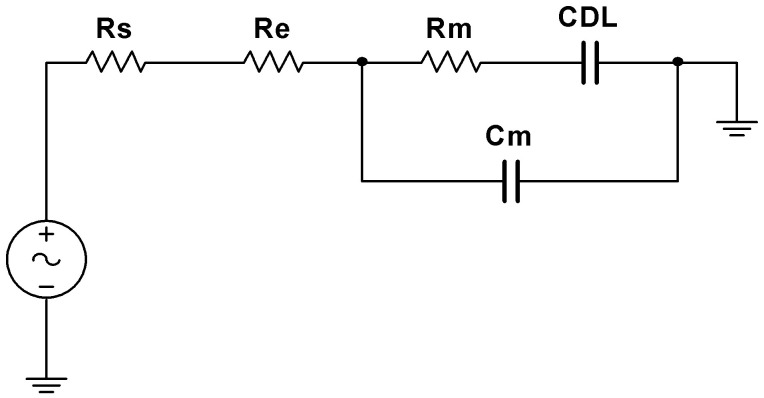
Equivalent electrical circuit model for the chip, with *R_e_* the electrode resistance, *R_m_* and *C_m_* the resistance and capacitance of the liquid medium and *C_DL_* the capacitance of the double layer.

To investigate the electric fields, double layer formation, and the heat generation as a function of the applied frequency, we built an equivalent electronic model (Figure 3) for the impedance. This model includes the resistance and capacitance of the liquid (*R_m_* and *C_m_*) and the capacitance of the double layer (*C_DL_*). *R_s_* is the voltage source resistance, which is about 50 Ohms. The resistance of the electrodes (*R_e_*) only becomes important when highly concentrated ionic solutions are used, with resistance comparable to that of the electrodes.

To estimate the values of *R_m_* and *C_m_* of the medium in a coplanar structure of electrodes, we use the following two approximate expressions (Supplementary Information I):

(1) $$R_{m}=\rho_{m}\frac{2\pi }{\mathrm{arcsinh}\left(u_{max}\right).l.n}$$

(2) $$C_{m}=\varepsilon_{0}\varepsilon_{m}\frac{\mathrm{arcsinh}\left(u_{max}\right).l.n}{\pi }$$

In these equations, $u_{max}$ is the ratio of distance between middle of two adjacent electrodes to the distance between them; *l* is the length of the electrodes and *n* is the number of electrodes. $\rho_{m}$ is the resistivity of the medium, $\varepsilon_{0}$ is the vacuum permittivity and $\varepsilon_{m}$ is the relative permittivity of the medium. The resistivity of the liquid $\rho_{m}$ can be provided by the supplier of the medium, it can be measured experimentally, but it can also be estimated from the ionic conductivity for a mixture of strong electrolytes [23]:

(3) $$\frac{1}{\rho_{m}}=\sigma_{I}=\sum n_{i}\lambda_{i}$$

with $\lambda_{i}$ representing the molar ionic conductivity for the ion with index *i* and $n_{i}$ the molar concentration. This formula is only applicable for low concentrations of ions when ionic interactions can be neglected [24].

The double-layer capacitance for low surface potentials (below 0.1 V) can be estimated from the Poisson–Boltzmann model [25]:

(4) $$C_{PB}=\varepsilon_{0}\varepsilon_{m}\lambda_{D}^{-1}\cosh\left(\frac{e\psi_{s}}{{2k}_{B}T}\right)l.w.n\;$$

where $\psi_{s}$ is a constant surface potential, $k_{B}$ is the Boltzmann constant and $\lambda_{D}$ the Debye–Hückel screening length:

(5) $$\lambda_{D}=\sqrt{\frac{\varepsilon_{0}\varepsilon_{m}k_{B}T}{2n_{b}e^{2}}}$$

In this equation, $2n_{b}$ is the total concentration (H^+^, OH^−^, K^+^ and Cl^−^) of ions per unit volume and *e* is the electron charge. This model is only applicable for low surface potential. Here we use a relatively high surface potential (0.5 V), apply the Stern model and include a Helmholtz capacitance in series [26]:

(6) $$\frac{1}{C_{DL}}=\frac{1}{C_{H}}+\frac{1}{C_{PB}}$$

with $C_{H}$ the Helmholtz layer capacitance:

(7) $$C_{H}=\frac{\varepsilon_{0}\varepsilon_{m}}{d_{H}}\;l.w.n$$

with *d_H_* the Helmholtz layer thickness (around 0.5 nm) [27].

The circuit model, including the above elements of the device (but without the internal resistance of the voltage source *R_s_*) with their estimated value, has been implemented in the NI Multisim 14.3 Education 14.3, and a frequency sweep from 10 Hz to 10 MHz has been executed to estimate the impedance of the circuit. This procedure has been repeated for four different concentrations of KCl in DI water, with the corresponding values of R_m_ and C_DL_.

### 2.3. Electric Field Calculations

**Figure 4:**
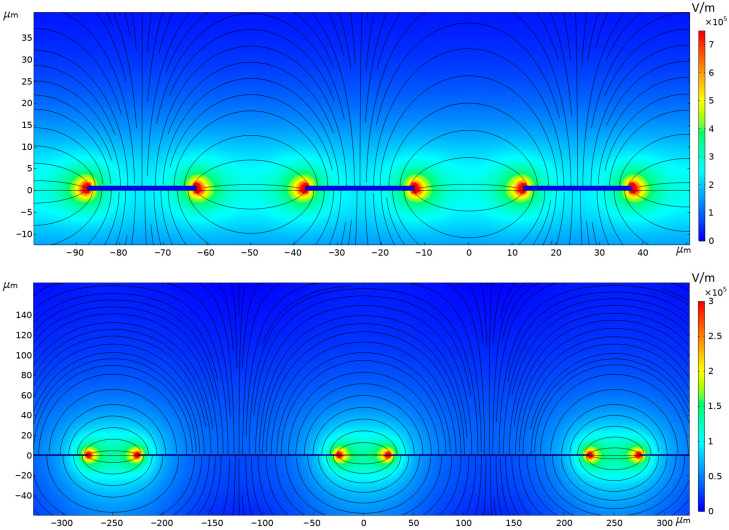
Electric field distributions (field lines and magnitude of the field) calculated for E25-S25 chip (**top**) and E200-S50 chip (**bottom**) when the sinusoidal applied voltage reaches its maximum value. The dielectric constant of water and glass are 80 and 4, respectively, and the conductivity of DI water is considered.

The electric field lines and the amplitude of the electric field vector in the coplanar electrode structures are simulated employing the commercial finite-element solver COMSOL Multiphysics 5.6, using the AC/DC module in time dependent study, as described in our previous work [22,28,29]. Two interdigitated ITO electrode configurations were simulated as shown in Figure 4. The first configuration (Figure 4, top) has 25 μm-wide electrodes with a gap of 25 μm (E25-S25 chip), whereas the second structure (Figure 4, bottom) has 200 μm width for the electrodes and 50 μm spacing between them (E200-S50 chip). In both cases, the thickness of the electrodes is 100 nm; however, a dark blue rectangle with height 1 μm is used in the figures to clearly indicate the location of the electrodes. A sinusoidal voltage signal (10 V peak amplitude) with variable frequency was applied across the electrodes. Even-numbered electrodes were connected to ground, while odd-numbered ones were connected to the sinusoidal voltage. A triangular mesh with “extra fine” element size was selected in the software to ensure accurate resolution (~0.1 μm near the edges of the electrodes). The simulations use the dielectric constants of water and glass, the conductivity of DI water, perfectly conductive electrodes, and periodic boundary conditions. The resulting electric field lines are shown in Figure 4 for both electrode structures.

### 2.4. Experimental Procedure

In the empty space between the two substrates, 50 mL of diluted medium with particles was added, and an AC voltage (3 V and 5 V peak sine waves) was applied over the interdigitated electrodes. The microchip was placed on an inverted microscope (Nikon Ti-E, Amstelveen, The Netherlands), and time-lapse imaging was performed using a 40× objective lens focused on the top surface of the chip to monitor particle accumulation near the electrodes. To analyze the particle motion under different electric frequencies, a custom MATLAB R2024b script was developed to count the number of particles in each frame of the recorded videos. The frames were first converted to binary (black and white) images through intensity thresholding, with the particles appearing as black spots against a white background. The number of particles (dark regions) was counted, using a minimum area criterion to filter out small artifacts and noise. This approach enables reliable particle detection and quantification across frames (Supplementary Information IV). This allowed us to assess particle accumulation as a function of frequency or applied voltage.

### 2.5. Electrical Impedance Measurement

To investigate the frequency-dependent behavior of double-layer formation, which influences key electrokinetic forces, we conducted impedance measurements across a broad frequency spectrum. These measurements enable the identification of critical frequency ranges where the impedance exhibits transitions, and the voltage division between the electrodes, the medium and the double layer is modified. For the measurements, we used a Hewlett Packard (HP) 4192ALF impedance analyzer, capable of measuring the real and imaginary part of the impedance (resistance and capacitance) over a frequency range from 5 Hz to 13 MHz, with an oscillation amplitude between 5 mV and 1.1 V.

**Figure 5:**
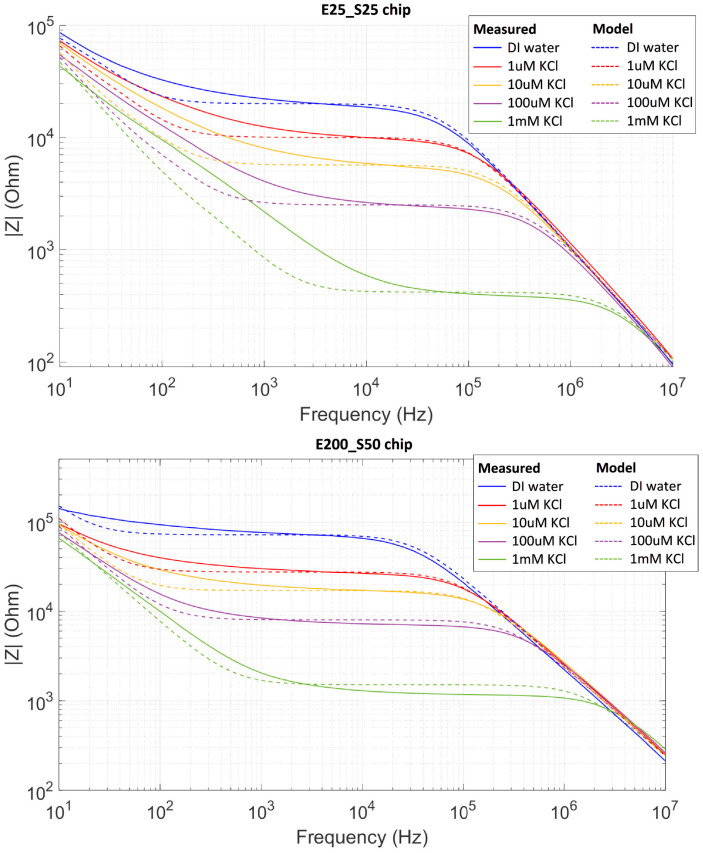
Impedance magnitude measurements (solid lines) and simulations based on an electrical model (dashed lines) for E25-S25 chip (**top**) and E200-S50 chip (**bottom**). The applied voltage is 1 V_p-p_ in both cases.

We investigated the electrical impedance of different aqueous solutions for the device with interdigitated electrodes as a function of the frequency of the applied AC voltage. The chips were filled with aqueous solutions with different concentrations of potassium chloride (KCl) salt. The measurements are shown in Figure 5 for a frequency range from 10 Hz to 10 MHz with amplitude 0.5 V. When polystyrene particles were added at low concentrations, the impedance measurements remained unchanged.

The impedance measurements reveal a strong dependence on salt concentration in the aqueous solution. The total impedance of the chip decreased significantly with increasing salt concentration, but the relation was not linear: a three-orders-of-magnitude increase in salt concentration (from 1 μM to 1 mM) resulted in approximately a hundred-fold decrease in the impedance. This is consistent with an increased ionic conductivity and a decreased Debye length at higher concentrations, which together lower the system’s overall resistance and capacitive impedance.

These measurements can be interpreted by using a simplified lumped-element equivalent circuit, in which the aqueous medium is characterized by three components: a resistor *R_m_*, a capacitor *C_m_* representing the bulk and a double layer capacitor *C_DL_* as illustrated in Figure 3. The circuit elements have been fitted, to match the experimental observation of the impedance versus frequency, leading to the values in Supplementary Information II.

The linear decreasing curve for high frequencies is independent of the ion concentration and represents the impedance of a constant capacitance due to the medium *C_m_* and the glass substrate [30]. The horizontal region in the impedance measurement around 10 kHz represents the sum of the resistances of the medium *R_m_* and the electrodes *R_e_*. Since K^+^ and Cl^−^ ions are the main contributors to the conductivity, the overall conductivity of the solution is directly related to the concentration of KCl. At low frequencies, the impedance decreases as frequency increases, which reflects the capacitive behavior of the diffuse double layer *C_DL_* near the electrode surface. In this regime, the applied field is largely screened in the bulk of the solution.

## 3. Results and Discussion

### 3.1. Electrokinetic Forces Acting on Particles

Before describing the experimental results, we first analyze the key electrokinetic forces in our system. In a liquid medium subjected to a non-uniform electric field, charged particles are subjected to three electrokinetic forces: electrophoresis (*EP*), dielectrophoresis (*DEP*), and electroosmosis (*EO*) [31]. For particles with low surface charge such as SiO_2_ microspheres, the electrophoretic (*EP*) contribution is weak compared to the dielectrophoretic (*DEP*) and electroosmotic (*EO*) effects. Although SiO_2_ particles are known to possess negatively charged silanol groups in aqueous media, the measured zeta potential in our experimental conditions was low (−20 mV, which is smaller than the −30 mV measured for carboxylated polystyrene particles). Furthermore, due to their higher density relative to water, SiO_2_ particles tend to sediment more rapidly [32], which reduces their apparent motion in the applied AC field and the effective electrophoretic mobility. In contrast, charged particles such as the polystyrene beads used in this study, experience all three forces, with the dominant one varying across different frequency ranges. Understanding these forces is essential to achieve controlled accumulation and manipulation of micro- and nanoparticles. Therefore, the electrode geometry and electrical parameters are tailored to enhance beneficial forces (primarily *DEP* and *EO*) while minimizing undesired effects. Additionally, Joule heating, which results from energy dissipation in the fluid, can induce convective flows that disrupt particle trapping. Recognizing these effects is critical for optimizing system performance and ensuring reliable results.

The **electrophoretic force *F****_EP_* for charged particles with charge *q* can be calculated by [22,33]:

(8) $$F_{EP}=qE$$

where ***E*** is the electric field vector for which the field lines are shown in Figure 4. The particles follow these field lines depending on their surface charge and electric field direction.

The **dielectrophoretic force *F****_DEP_* is given by [34]:

(9) $$F_{DEP}=2\pi r_{p}^{3}\varepsilon_{0}\varepsilon_{m}\;Re\left[CM\left(\omega \right)\right]\nabla {\left|E\right|}^{2}$$

where $r_{p}$ is the particle radius, $\varepsilon_{0}$ is the vacuum permittivity, $\varepsilon_{m}$ is the relative permittivity of the liquid medium around the particles, $\nabla {\left|E\right|}^{2}$ is the gradient of the squared electric field magnitude, and $CM\left(\omega \right)$ is the Clausius-Mossotti factor which can be written as:

(10) $$CM\left(\omega \right)=\frac{\varepsilon_{p}^{*}-\varepsilon_{m}^{*}}{\varepsilon_{p}^{*}+2\varepsilon_{m}^{*}}$$

where $\varepsilon_{p}^{*}=\varepsilon_{p}-\frac{j\sigma_{p}}{\omega }$ and $\varepsilon_{m}^{*}=\varepsilon_{m}-\frac{j\sigma_{m}}{\omega }$ represent the complex permittivity of the particle and the medium, respectively. Here, $\varepsilon_{p}=2.9\varepsilon_{0}$ and $\varepsilon_{m}=80\varepsilon_{0}$ are the real part of the permittivities and $\sigma_{p}=10^{-19}\;S$ and $\sigma_{m}$ are the conductivities. At high frequencies, the imaginary parts of the complex permittivity ($\frac{j\sigma }{\omega }$) become negligible compared to the real part (ε). Because the permittivity of water (~80 $\varepsilon_{0}$) is much higher than that of the particles (~2.9 $\varepsilon_{0}$), the Clausius–Mossotti factor then becomes negative, causing particles to experience negative dielectrophoresis (i.e., motion toward low-field regions).

At low frequencies, however, the imaginary term becomes comparable in magnitude to the real part, resulting in a frequency-dependent behavior of the CM factor. There is a crossover frequency where the real part of the CM factor changes sign. The sign of this factor is critical, as it determines the direction of dielectrophoretic motion:

- *R_e_* [*CM*(*ω*)] > 0: Positive DEP (pDEP)—particles move toward high-field regions.
- *R_e_* [*CM*(*ω*)] < 0: Negative DEP (nDEP)—particles move toward low-field regions.

**Figure 6:**
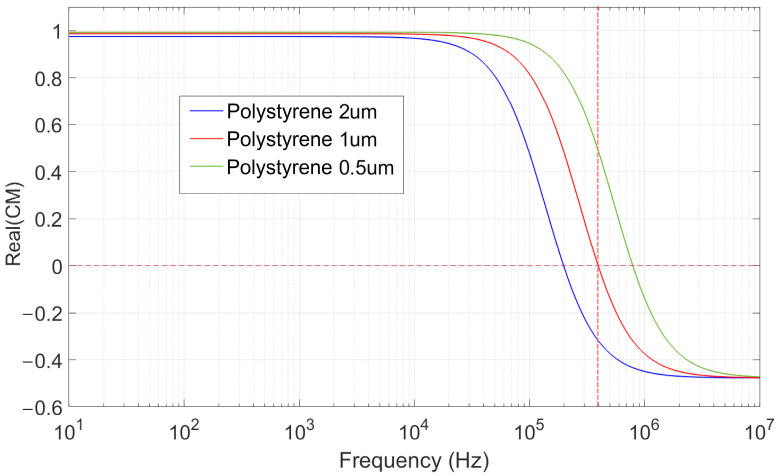
Clausius–Mossotti factor for dielectrophoresis, for three different particle sizes, in deionized water. The vertical red dashed line indicates 400 kHz.

The electric conductivity of the medium can be calculated or measured, and the equivalent conductivity of the particles can be estimated from this formula:

(11) $$\sigma_{p}=\sigma_{bulk}+\frac{2K_{s}}{r_{p}}$$

where *K_s_* is the surface conductivity of the particles (Equation (12)) and $\sigma_{bulk}$ = 1 × 10^−16^ S/m is the bulk conductivity of polystyrene material which is negligible [35]. The calculated CM factor estimated with MyDEP app 1.0.1 [36] is shown in Figure 6. As depicted, the real part of the CM factor is positive (~1) for all studied particles in this article for frequencies below about 100 kHz. But for frequencies between ~200 kHz and 1 MHz, zero-crossing transitions happen. Above 2 MHz, the real part of the CM factor becomes negative for all particles.

As the ionic concentration of the surrounding electrolyte increases, the surface conductivity ($K_{s}$) of charged particles rises due to enhanced ion density in the diffuse layer. For a charged polystyrene particle, $K_{s}$ can be calculated based on the Bikerman model [37]:

(12) $$K_{s}=\frac{4F_{F}^{2}z^{2}D\sqrt{n_{b}}\left(1+\frac{3m}{z^{2}}\right)}{R_{g}T\sqrt{\frac{2e^{2}}{\varepsilon_{0}\varepsilon_{m}k_{B}T}}}\;\left(\cosh\frac{zF_{F}\zeta }{2R_{g}T}-1\right)$$

Here, the parameter *m* characterizes the contribution of electro-osmosis to the motion of ions within the double layer:

(13) $$m=\frac{2\varepsilon_{0}\varepsilon_{m}R_{g}^{2}T^{2}}{3\eta F_{F}^{2}D}$$

where *F_F_* is the Faraday constant, *T* is the absolute temperature, *R_g_* is the gas constant, $n_{b}$ is the ionic concentration in the bulk fluid, *z* is the ion valency, *ζ* is the electrokinetic potential (particle zeta potential), *D* = 2.5 × 10^−13^ m^2^/s is the PS particle (1 μm diameter) diffusion coefficient in water [38], and η=1 mPa.s is the dynamic viscosity of the liquid. In our case, *K_s_* = 2 × 10^−9^ S with the mentioned parameters.

**Figure 7:**
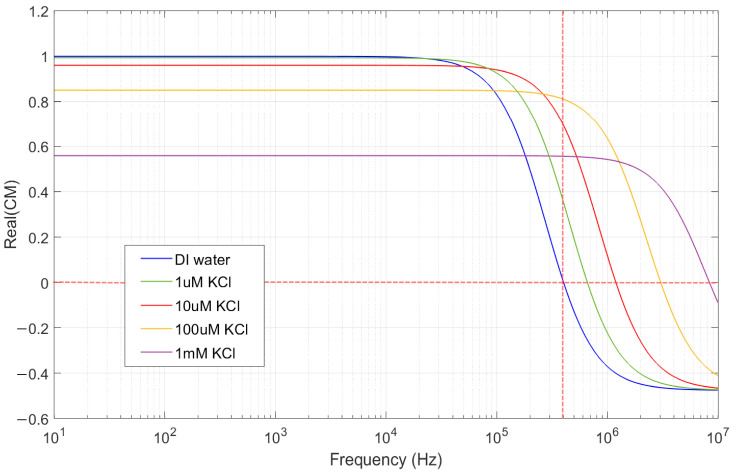
Clausius-Mossotti factor for dielectrophoresis, for 1 μm PS particle with different KCl concentrations. The vertical red dashed line indicates 400 kHz.

Equation (12) shows that $K_{s}$ increases sub linearly ($\propto \sqrt{n_{b}}$) with ionic strength, particularly in the range of 1 μM to 1 mM KCl. This increase in surface conductivity leads to a higher effective electrical conductivity of the particle ($\sigma_{p}$), which in turn shifts the zero-crossing frequency of the Clausius–Mossotti factor to higher values, as depicted in Figure 7. However, the ionic conductivity of the surrounding medium, $\sigma_{m}$, also increases substantially with the salt concentration. Since the CM factor depends on the contrast between $\sigma_{p}$ and $\sigma_{m}$, a simultaneous increase in both parameters results in a reduction in the magnitude of the CM factor. The frequency dependency based on Equation (12) is illustrated in Figure 7 for three different KCl concentrations.

The **thermal convection force F_TC_** within the medium above the electrodes cannot be calculated directly. However, for deionized (DI) water, the current passing through the medium at frequencies below 1 MHz is very small (~10 µA). Consequently, the power generated in the chip may be considered negligible. In cases with significant Joule heating, such as when the ion concentration is high, the liquid density can change, potentially leading to strong buoyancy-driven convection [39,40]. This type of flow can be regarded as part of the electrothermal velocity component ***v_ET_***.

By using a low conductivity medium (DI water) and applying AC voltage to the chip, the ions in the double layer formed on the electrodes will migrate and due to the viscosity, the solution surrounding the ions will also move, creating an **Electroosmotic slip velocity**. Under low applied potentials and negligible electrode polarization, nonlinear distortions of the double layer can be ignored, and the added complexity of the full AC electroosmosis theory offers little practical advantage. This leads to the Helmholtz–Smoluchowski velocity [41]:

(14) $$v_{EO}=-\frac{\varepsilon_{m}}{\eta }\zeta_{DL}\;E_{x}$$

where *ζ_DL_* is the double layer zeta potential, ***E****_x_* is the tangential electric field in the double layer, and *η* is the dynamic viscosity of the medium. Note that for ac voltage driving, there may be a considerable net electro-osmotic velocity, because of the intricate interaction between the electric field and the dynamics of the distribution in the double layer.

The **hydrodynamic drag** for large particles with radius *r_p_* due to the convection force is:

(15) $$F_{drag}=-6\pi r_{p}\eta (v_{p}-v_{m})$$

where ***v****_p_* is the particle velocity and ***v****_m_* is the local fluid velocity, which includes contributions from both electrothermal ***v_ET_*** and electroosmotic flow ***v_EO_***. For more complex geometries, hydrodynamic interactions, or wall effects, the drag can be generalized using the hydrodynamic resistance tensor ***R_H_*** [42]:

(16) $$F_{drag}=-R_{H}(v_{p}-v_{m})$$

The total force acting on the particles can be calculated as a sum of all these contributing forces [43]:

(17) $$F_{tot}=\frac{m_{p}dv_{p}}{dt}=F_{EP}+F_{DEP}+F_{drag}$$

If inertial effects are negligible compared to viscous drag, the left-hand side can be approximated as zero:

(18) $$R_{H}\left(v_{p}-v_{m}\right)=F_{EP}+F_{DEP}$$

Thus, the final expression for the particle velocity is:

(19) $$v_{p}=v_{bf}+v_{EO}+v_{ET}+\frac{F_{EP}}{R_{H}}+\frac{F_{DEP}}{R_{H}}$$

where $v_{bf}$ is the background fluid velocity.

Based on these forces and velocities, we can selectively enhance or suppress specific mechanisms across different frequency ranges. For example, at low frequencies, *EO* and *EP* tend to dominate, while at higher frequencies, *DEP* becomes more significant as *EO* weakens due to reduced double-layer polarization. The aim is to tune the system parameters, including voltage amplitude, frequency, and ionic concentration, to control which force dominates as illustrated in Figure 6 for particles with different size. This tunability is key for size-based sorting and selective particle trapping, offering a flexible approach that does not depend on particle material composition.

### 3.2. Electrokinetic Motion of the Particles in DI Water

We investigate the motion of polystyrene particles diluted in deionized water (>1 MOhm.cm) under influence of an AC electric field. As mentioned, in DI water (pH ~6) where the conductivity is low and the impedance of the chip is high (Figure 5), the convective force as well as the heat generation on the electrodes and between them is negligible. Under these slightly acidic conditions (pH 5.5–6), the carboxyl groups on the surface of the polystyrene particles are only partially deprotonated, resulting in a relatively weak negative surface charge. This leads to a relatively weak electrophoretic force. Hence, the effective forces in low conductivity media such as DI water are mainly *EO* and *DEP*.

**Figure 8:**
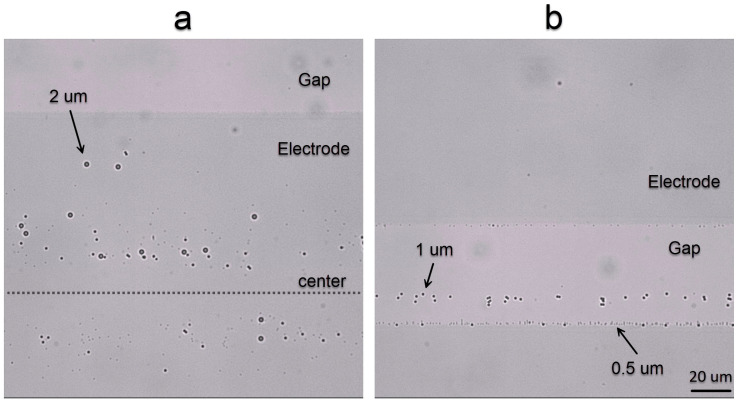
Accumulation of particles with different diameters (0.5, 1 and 2 μm), for two different frequencies (**a**) 1 kHz and (**b**) 400 kHz with amplitude 3 V in the E200-S50 chip, after a time interval of 10 s.

For the first experiment, the E200-S50 chip is used. The darker areas in Figure 8 represent the ITO electrodes, and the lighter areas are the space between them (gap). In this experiment (Video S1 in Supplementary Information V), all particles are attracted to the middle of the electrodes when 100 Hz to 10 kHz is applied with 3 V of amplitude for 10 s (Figure 8a). When a frequency above 200 kHz is applied, 0.5 μm particles are attracted to the edges of the electrodes (Figure 8b), 1 μm particles to the middle of the gap (Figure 8b) and 2 μm particles are pushed away from the electrodes. For frequencies between 10 kHz and 200 kHz, the accumulation of particles is less prominent in this chip.

**Figure 9:**
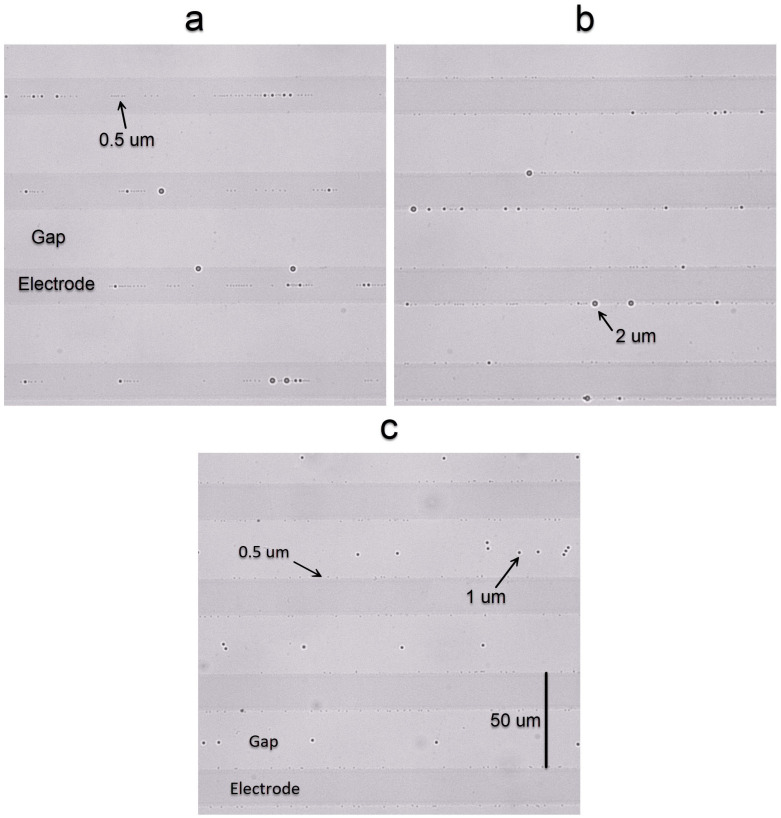
Particle accumulation in three different frequencies (**a**) 1 kHz, (**b**) 50 kHz and (**c**) 400 kHz and 3 v in E25-S25 chip after 10 s.

In another set of experiments (Video S2 in Supplementary Information V), we used the E25-S25 chip with 3 V of applied voltage for 10 s. Similarly to the E200-S50 chip, for frequencies < 10 kHz, all particles accumulate in the middle of the electrodes (Figure 9a). Instead, for frequencies between 10 kHz and 100 kHz, all particles were attracted to the edges of electrodes (Figure 9b). By increasing the frequency to 400 kHz, we achieved a complete separation of 3 sizes of particles (Figure 9c): 0.5 μm particles are attracted to the edges of the electrodes, 1 μm particles accumulate in the middle of the gap and 2 μm particles move away from the top surface with electrodes.

In the very low-frequency range, typically between 20 Hz and 800 Hz, electroosmotic (*EO*) flow becomes the dominant driving mechanism for particle motion (see Video S3 in Supplementary Information V) [41]. In this regime, the ions within the diffuse layer of the electrical double layer migrate in response to the surface-parallel (tangential) component of the oscillating electric field, as described by Equation (14). Due to the asymmetry in the distribution and mobility of these ions near the electrode surface, their movement generates a net time-averaged fluid flow, known as AC electroosmosis. This flow typically exhibits a recirculating pattern concentrated near the electrode edges, where the electric field gradients are strongest [44].

Although dielectrophoresis (*DEP*) also influences the particles, pulling them toward regions of high electric field intensity (see Figure 6), its effect is significantly weaker than that of *EO* under these conditions. As a result, the motion of suspended particles is primarily governed by the EO-induced fluid circulation, which transports them along streamlines that swirl around the electrode edges (see Video S3 in Supplementary Information V). Such motion interferes with the localized accumulation of particles and is thereby intentionally avoided (Supplementary Information III).

In the low frequency range (1 kHz to 10 kHz), the influence of the dielectrophoretic force (*DEP*) is more pronounced. A balance between *EO* and *DEP* will establish at the middle of electrodes (Figure 8a and Figure 9a), leading to a temporary accumulation of particles in that region. This phenomenon is observed in both E200 and E25 chips (Figure 8a and Figure 9a) and occurs consistently across all particle sizes. This is because the *DEP* remains positive for all particles (Figure 6), while the electroosmotic flow, which drives the bulk fluid motion, affects them almost similarly regardless of their size.

At mid-range frequencies (10 kHz to 200 kHz), the electroosmotic velocity becomes negligible [45], and the dielectrophoretic force dominates over the particle motion. As *DEP* remains positive for all studied particle sizes (500 nm, 1 μm, and 2 μm) shown in Figure 6, they are attracted toward the regions of highest electric field intensity, located at the electrode edges (Figure 9b).

In the high-frequency range (above 1 MHz), the CM factor of the particles crosses zero (Figure 6), indicating a change in the sign of the *DEP* force. As a result, particles are repelled from regions with high electric field intensity, i.e., the electrodes. According to Figure 6, the first zero-crossing occurs for the 2 μm particles at approximately 200 kHz. Beyond this frequency, these particles experience negative *DEP* and are pushed away from the electrodes. Interestingly, at the zero-crossing frequency, a quasi-equilibrium region forms at the center of the gap between the electrodes [41], where particles tend to accumulate temporarily. At 400 kHz, the CM factor is negative for 2 μm particles, approximately zero for 1 μm particles, and positive for 500 nm particles. Consequently, the 2 μm particles are repelled into the bulk solution, the 1 μm particles accumulate near the center of the electrode gap, and the 500 nm particles are attracted toward the electrode edges. This behavior is clearly confirmed by the experimental observations shown in Figure 8b and Figure 9c.

Although this study focuses on standard polystyrene micro-particles, the same electrokinetic principles can be extended to biological cells. The frequency-dependent dielectrophoretic behavior can be predicted by evaluating the Clausius–Mossotti factor for specific cell types, using the same analytical framework (Equations (9)–(13)) or MyDEP simulations. Considering the typical cell size (~10 μm) and dielectric properties, appropriate frequency tuning could enable selective trapping or sorting of biological cells. Furthermore, to enable a practical implementation of the proposed technique, the chip design can be further integrated with a microfluidic channel positioned above the interdigitated electrodes. This configuration allows the separated or accumulated particles to be transported along the electrode array and collected at designated outlets by applying a controlled flow. Such integration would transform the current proof-of-concept device into a fully functional on-chip separation system, combining precise electrokinetic control with efficient particle collection.

### 3.3. Electrokinetic Particle Motion for Different Salt Concentrations

Next, we investigate the effect of the ion concentration (KCl) in the medium on the particle motion and accumulation. The frequency and amplitude of the applied voltage was 1 kHz and 5 V, respectively, to be sure that the accumulation of particles will happen at the center of the electrodes, and four concentrations were tested using the E200-S50 IDEs and 1 μm particles. We specifically used this chip to decrease joule heating on the electrodes and its disruptive effects on the particle accumulation in high concentration of ions.

**Figure 10:**
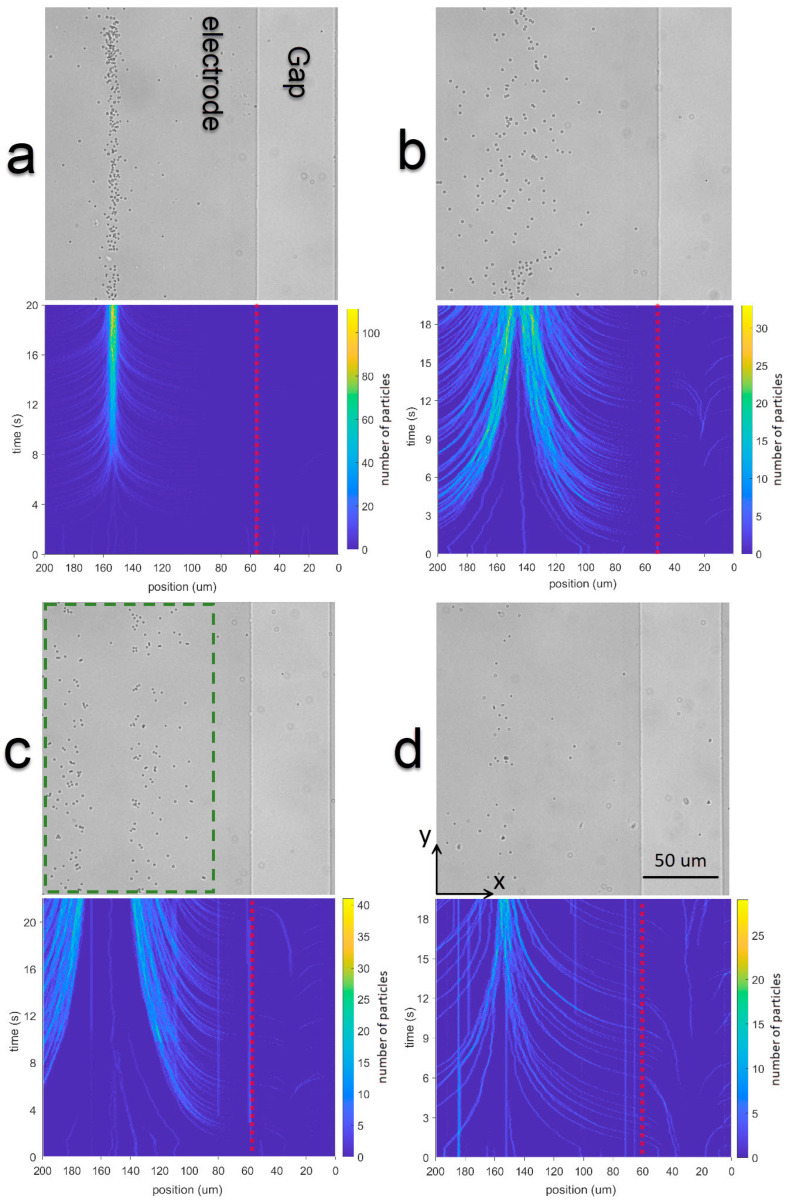
Microscopy images of the accumulation of polystyrene particles (diameter 1 μm), after a 5 V peak sine voltage of 1 kHz during 20 s in a E200-S50 chip, using 4 different concentrations of KCl in DI water. Corresponding histograms are shown below each image of the total number of particles along y as a function of the position x and time. (**a**) 1 mM KCl, (**b**) 10 mM KCl, (**c**) 100 mM KCl, and (**d**) 1 mM KCl. Red dashed line is the edge of the electrode.

Particle motion and accumulation was observed in time-lapse microscopy videos using a 40× objective lens (Figure 10). Video frames are analyzed to integrate the number of particles in the *y*-direction and show the distribution of particles as a function of *x*, real position of the electrode and the gap.

**Figure 11:**
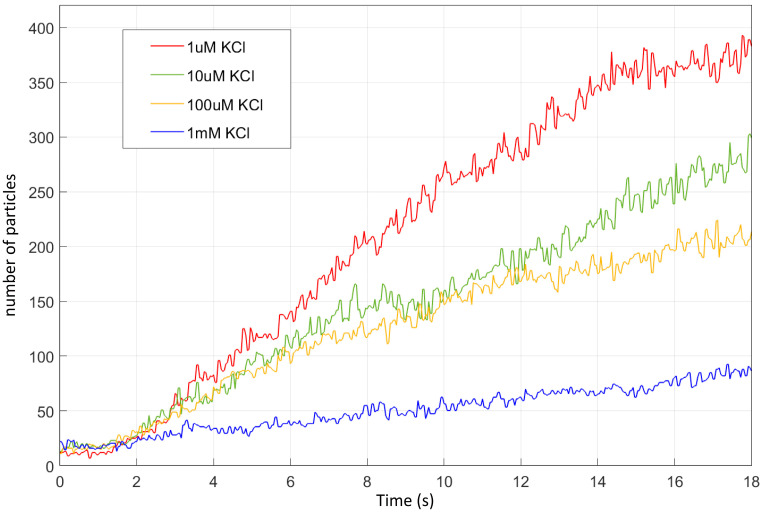
Particle accumulation on the electrode over 18 s following the application of 5 V peak sine voltage of 1 kHz in the E200-S50 chip (inside the dashed green area shown in Figure 10c).

In the frequency range used in this set of experiments (1 kHz), which falls within the low frequency range, a balance between *EO* and *DEP* is established at the center between the electrodes (see Video S4 in Supplementary Information V). As a result, for the lowest concentration of KCl, the polystyrene particles migrate toward the electrode center (Figure 10a). At higher KCl concentrations (higher conductivity), fewer particles are observed near the electrodes (Figure 10d) and the migration toward the center becomes slower (Figure 11). Nevertheless, at 1 kHz, particles generally tend to accumulate at the center of the electrodes, but the screening effect and the convective force will attenuate this attraction and less particles will be accumulated (Figure 10).

It is worth noting that at lowest concentration of KCl (1 μM), the contribution of intrinsic ions from water dissociation (H^+^ and OH^−^) becomes comparable to that of the added salt. The estimated concentrations of these ions in deionized water at pH ≈ 6 are on the order of 1 μM for H^+^ and OH^−^, which are not negligible relative to 1 μM KCl. Therefore, water dissociation partially contributes to the overall ionic strength of the medium and affects the formation of the electric double layer. However, because the local electrokinetic response is primarily governed by the electrode geometry and the applied AC field amplitude, the additional ionic species do not significantly alter the observed particle trajectories under our experimental conditions.

At high ionic concentrations, enhanced screening effects suppress the interaction between the particles and the applied electric field, thereby reducing the effectiveness of electrokinetic forces and inhibiting particle accumulation [46]. Moreover, an increased ionic strength raises the solution’s conductivity, resulting in higher current flow through the chip and, consequently, more pronounced Joule heating, particularly near the electrode edges. As the salt concentration increases, the crossover frequency of the Clausius–Mossotti factor shifts toward higher values. Although this shift does not significantly affect the frequency range used in this part of our experiments (1 kHz), the reduction in the real part of the CM factor leads to a decrease in the magnitude of the dielectrophoretic (*DEP*) force (Figure 7). This reduction can limit the efficiency of particle manipulation efficiency at higher salt concentrations.

Figure 11 quantitatively illustrates this trend by showing the time-dependent accumulation of particles near the center of the E200-S50 chip over 18 s for varying KCl concentrations. At low ionic strength (1 μM KCl), particle accumulation is both rapid and substantial, reflecting strong and unopposed electrokinetic forces. As the KCl concentration increases, the accumulation rate decreases. This reduction is attributed to increased double-layer screening, which limits the effective field penetration and weakens *EO* and *DEP* forces. Moreover, higher conductivity enhances current flow, leading to Joule heating at the electrode edges and promoting convective flows that oppose accumulation. Thus, these results highlight the importance of controlling ionic strength for efficient and selective electrokinetic manipulation of nanoparticles.

## 4. Conclusions

In this study, electrokinetic motion of charged polystyrene particles in an aqueous solution near interdigitated electrodes was investigated. We demonstrate that, for any mixture of particles in DI water, a specific frequency range can be identified to spatially separate the particles (here as a function of size). By using known particle properties to calculate their CM factor, this optimal frequency can be determined. However, in ion-rich solutions, this separation effect can be disrupted due to the screening effect and convective flows in the liquid. These results highlight the critical role of ionic strength in tuning electrokinetic behavior and optimizing particle manipulation in microfluidic systems. By systematically varying the KCl concentration, we demonstrate that even subtle changes in conductivity can significantly impact particle accumulation dynamics, due to modulation of electric field screening and thermally induced convection. This insight is particularly valuable for designing low-cost, label-free platforms for particle sorting or enrichment, where precise control over electrokinetic forces is essential. Moreover, the ability to manipulate particles of identical material composition purely based on environmental conditions opens new avenues for applications in biosensing, diagnostics, and environmental monitoring.

## Data Availability

Data available in a publicly accessible repository.

## Supplementary Materials

The following supporting information can be downloaded at: https://www.mdpi.com/article/10.3390/nano15201568/s1, Figure S1: Conformal transformation; Figure S2: polystyrene particles circulation at 100 Hz in E25-S25 chip; Figure S3: converting process of video frames to binary pictures; Table S1: Simulated fitted values for all components of the circuit model, E200-S50 chip with different concentration of KCl in DI water; Table S2: Simulated fitted values for all components of the circuit model, E25-S25 chip with different concentration of KCl in DI water; Video S1: Accumulation of particles with different diameters (0.5, 1 and 2 μm), for two different frequencies in the E200-S50 chip; Video S2: Accumulation of particles with different diameters (0.5, 1 and 2 μm), for three different frequencies in the E25-S25 chip; Video S3: Particles circulation above the electrodes at 100 Hz in E25-S25 chip; Video S4: Accumulation of 1 μm particles with four different concentration of KCl at 1 kHz in the E200-S50 chip, etc. (https://doi.org/10.5281/zenodo.17153131, accessed on 18 September 2025).

## Abbreviations

The following abbreviations are used in this manuscript:

ITO	Indium tin oxide	
IDE	Interdigitated electrodes	
AC	Alternating current	
DC	Direct current	
DI	Deionized	
*DEP*	Dielectrophoresis	
*EP*	Electrophoresis	
*EO*	Electroosmosis	
TC	Thermal convection	
List of Symbols		
Symbol	Explanation	Amount
*R_m_*	Resistance of the liquid on the electrodes inside the channel	-
*C_m_*	Capacitance of the liquid on the electrodes inside the channel	-
*u_max_*	The ratio of distance between middle of two adjacent electrodes to the distance between them	-
*ρ_m_*	Resistivity of the medium	-
*ε_0_*	Vacuum permittivity	8.854 × 10^−12^ (F/m)
*ε_m_*	Relative permittivity of the medium	-
*l*	Electrode length	-
*n*	Number of electrodes	-
*w*	Electrode width	-
*A*	Area of electrodes (w × l)	-
*n_i_*	Number of ions i in the compound in the medium (e.g., salts)	-
*n_b_*	Concentration of compound (e.g., salts)	-
*λ_i_*	Molar ionic conductivity for ion i	-
*σ_i_*	Total ionic conductivity of the medium	-
*C_DL_*	Double layer capacitance	-
*C_PB_*	Poisson–Boltzmann model capacitance of double layer	-
*C_H_*	Helmholtz layer capacitance	-
*λ_d_*	Debye–Hückel screening length	-
*k_B_*	Boltzmann constant	1.38 × 10^−23^ (m^2^ kg^2^/s^2^ K)
*e*	Electron charge	1.602 × 10^−19^ C
*ψ_s_*	Constant surface potential	-
*d_H_*	Helmholtz layer thickness	-
*r*, *r_p_*	Particle radius	-
*CM*	Clausius–Mossotti factor	-
*T*	Absolute temperature in Kelvin	298 K
$\varepsilon_{m}^{*}$	Complex permittivity of medium	-
$\varepsilon_{p}^{*}$	Complex permittivity of particle	-
*ε_m_*	Relative permittivity of medium (water)	80
*ε_p_*	Relative permittivity of particle (polystyrene)	2.9
*σ_m_*	Conductivity of medium	-
*σ_p_*	Conductivity of particle	~1 × 10^−19^ S
*ω*	Angular frequency (2πf)	-
*K_s_*	Surface conductivity of the particle	-
*F_F_*	Faraday constant	96,485.3 (s.A/mol)
*R_g_*	Gas constant	8.314 (J/mol.K)
*z*	Ion valency	-
*D*	Particle diffusion coefficient	-
*κ*	Inverse Debye–Hückel length	-
*η*	Dynamic viscosity of the liquid	-
*ζ*	Electrokinetic potential	30 mV
*ζ_dl_*	Double layer zeta potential	-
***v****_p_*	Particle velocity	-
***E***	Electric field	-
***F****_EP_*	Electrophoretic force	-
***F****_DEP_*	Dielectrophoretic force	-
***F****_TC_*	Thermal convection force	-
***F****_EO_*	Electroosmotic force	-
***F****_drag_*	Drag force	-
***v****_EO_*	Electroosmotic velocity	-
***v****_ET_*	Electrothermal velocity	-
