# Supplementary material for: Tunable Electrokinetic Motion of Charged Nanoparticles in an Aqueous Solution Using Interdigitated Microelectrodes
Source: Nanomaterials (Basel). 2025 Oct 15;15(20):1568. doi: 10.3390/nano15201568 (PMC12566736; doi:10.3390/nano15201568)
Supplement: Supplementary file 1 [file nanomaterials-15-01568-s001.zip › nanomaterials-3910846-supplementary.pdf]

Supplementary Information I

Field calculation for two planar electrodes with gap d

Conformal transformation z=sin(w).

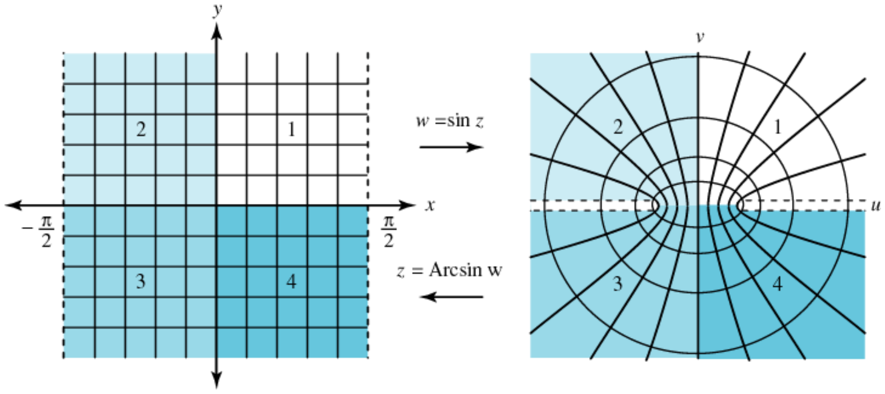

Figure S1 conformal transformation
z=x+iy= u+iv

In this transformation we have:

$$\begin{cases} w = \sin(z) \\ u + iv = \sin(x + iy) \end{cases}$$

We assume that the capacitance is infinite (out-of-plane thickness).

In our electrodes, the y coordinate is limited to the value corresponding to the middle of the electrode with x=pi/2.

The distance between the middles of neighboring electrodes defines "u_{max} = distance between middles of electrodes / distance between electrodes". In our case, u_{max} can have 2 values:

$$u_{max} = \frac{w+s}{s} \to \begin{cases} E200 - S50\ chip \to\ u_{max} = \frac{200\mu m + 50\mu m}{50\mu m} = 5 \\ E25 - S25\ chip \to\ u_{max} = \frac{25\mu m + 25\mu m}{25\mu m} = 2 \end{cases}$$

At the surface where the electrodes are we have v=0, x=pi/2. So, to calculate y:

$$\frac{u^2}{\sinh^2(y)} + \frac{v^2}{\cosh^2(y)} = 1 \overset{v=0}{\Longrightarrow} \frac{{u_{max}}^2}{\sinh^2(y)} = 1 \to {u_{max}}^2 = \sinh^2(y)$$

$$y = \operatorname{arcsinh}(u_{max})$$

So, if we now name the out-of-plane thickness as l (length of electrodes), we will have:

$$C = \frac{\varepsilon_0 \varepsilon_r A}{d} \xrightarrow[d=\pi]{A_{top}=y.l \quad A_{bottom}=y.l} C = \varepsilon_0 \left(\varepsilon_{r_{top}} + \varepsilon_{r_{bottom}}\right) \frac{\operatorname{arcsinh}(u_{max}).\,l}{\pi}$$

This also can be used for resistance:

$$R = \rho \frac{l}{A} \to R = \rho \frac{2\pi}{\operatorname{arcsinh}(u_{max}).\,l}$$

This would be for one pair of electrodes and for all electrodes, it should be divided by n (number of electrodes) for resistance and multiplied by n for capacitance.

Supplementary Information II

Concentration / Component	DI water	1 μM	10 μM	100 μM	1 mM
C_{DL}	100 nF	120 nF	150 nF	180 nF	220 nF
C_m	65 pF	65 pF	65 pF	65 pF	65 pF
R_m	120 kΩ	40 kΩ	25 kΩ	8 kΩ	1.5 kΩ

Table S1. Simulated fitted values for all components of the circuit model, E200-S50 chip with different concentration of KCl in DI water.

Concentration / Component	DI water	1 μM	10 μM	100 μM	1 mM
C_{DL}	100 nF	120 nF	140 nF	160 nF	190 nF
C_m	130 pF	130 pF	130 pF	130 pF	130 pF
R_m	25 kΩ	12 kΩ	7 kΩ	3 kΩ	500 Ω

Table S2. Simulated fitted values for all components of the circuit model, E25-S25 chip with different concentration of KCl in DI water.

Supplementary Information III

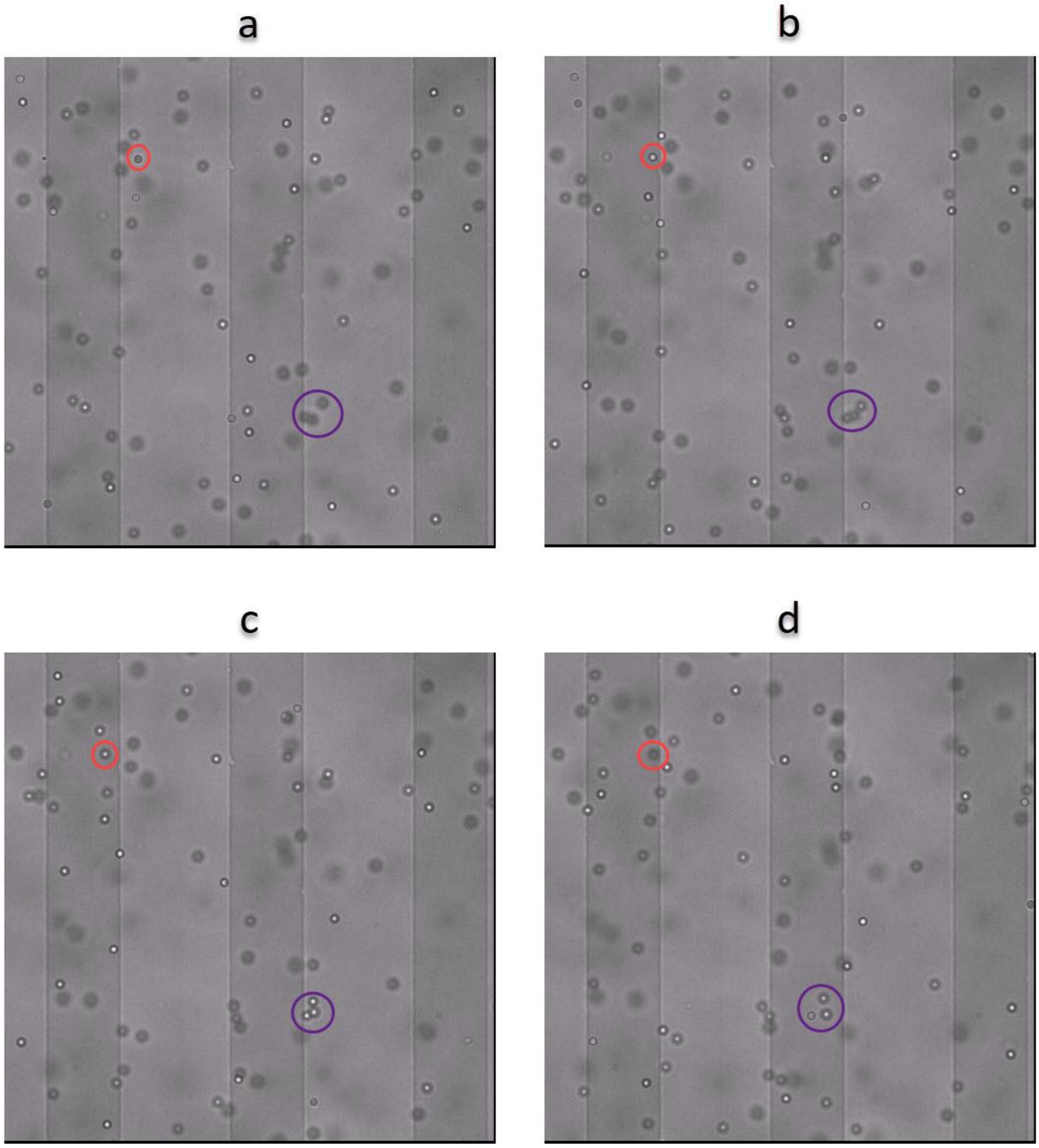

Figure S2: polystyrene particles circulation at 100 Hz in E25-S25 chip

Polystyrene particles (diameter 1 µm) circulation above the electrode edges microscope images, after a 3V peak sine voltage of 100 Hz during 20 s in a E25-S25 chip. Circles (red and purple) show particles that comes in focus (near the surface) then move to the middle of electrodes and then pushed away from the surface.

Supplementary Information IV

An example of the process of converting the video frames to binary pictures to be able to count the particles in each frame:

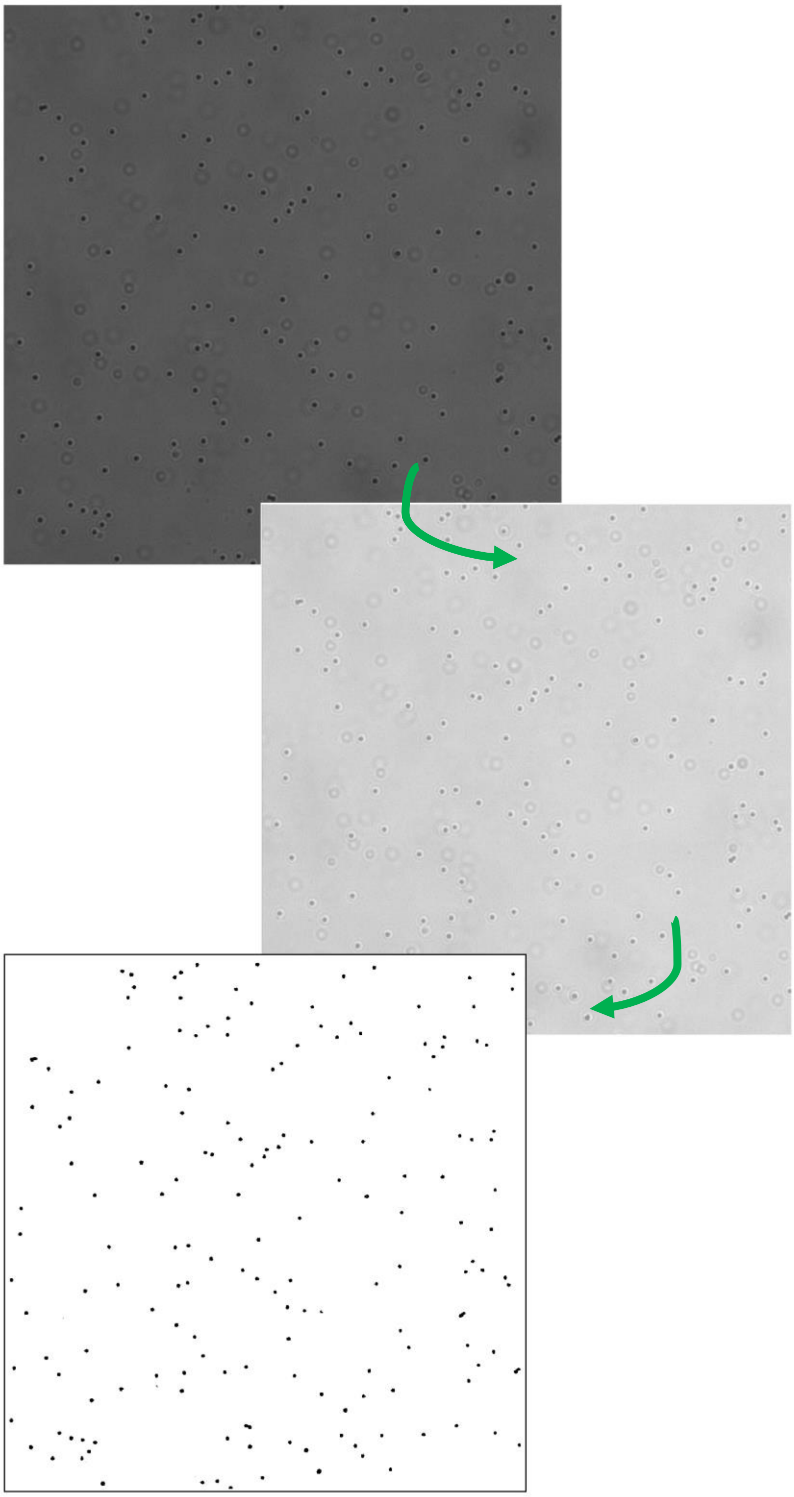

Figure S3: converting process of video frames to binary pictures

Supplementary Information V

Supplementary videos for the "Tunable electrokinetic motion" manuscript are available at the following link:

https://doi.org/10.5281/zenodo.17153131
